# Effects of Frequency Discrimination Training on Tinnitus: Results from Two Randomised Controlled Trials

**DOI:** 10.1007/s10162-012-0323-6

**Published:** 2012-04-04

**Authors:** Derek J. Hoare, Victoria L. Kowalkowski, Deborah A. Hall

**Affiliations:** 1 NIHR National Biomedical Research Unit in Hearing, Ropewalk House, 113 The Ropewalk, Nottingham, NG1 5DU UK; 2 School of Clinical Sciences, The University of Nottingham, Nottingham, NG7 2RD UK; 3 Division of Psychology, School of Social Sciences, Nottingham Trent University, Burton Street, Nottingham, NG1 4BU UK

**Keywords:** chronic tinnitus, tinnitus handicap, perception, loudness, plasticity

## Abstract

That auditory perceptual training may alleviate tinnitus draws on two observations: (1) tinnitus probably arises from altered activity within the central auditory system following hearing loss and (2) sound-based training can change central auditory activity. Training that provides sound enrichment across hearing loss frequencies has therefore been hypothesised to alleviate tinnitus. We tested this prediction with two randomised trials of frequency discrimination training involving a total of 70 participants with chronic subjective tinnitus. Participants trained on either (1) a pure-tone standard at a frequency within their region of normal hearing, (2) a pure-tone standard within the region of hearing loss or (3) a high-pass harmonic complex tone spanning a region of hearing loss. Analysis of the primary outcome measure revealed an overall reduction in self-reported tinnitus handicap after training that was maintained at a 1-month follow-up assessment, but there were no significant differences between groups. Secondary analyses also report the effects of different domains of tinnitus handicap on the psychoacoustical characteristics of the tinnitus percept (sensation level, bandwidth and pitch) and on duration of training. Our overall findings and conclusions cast doubt on the superiority of a purely acoustic mechanism to underpin tinnitus remediation. Rather, the nonspecific patterns of improvement are more suggestive that auditory perceptual training affects impact on a contributory mechanism such as selective attention or emotional state.

**Electronic supplementary material:**

The online version of this article (doi:10.1007/s10162-012-0323-6) contains supplementary material, which is available to authorized users.

## Introduction

Tinnitus is likely to arise from abnormal neural activity in the auditory pathway which is erroneously interpreted by the brain as an acoustic signal (Jastreboff 1990). The primary cause of tinnitus is thought to be hearing loss. Neurons in the central auditory system are deprived of their normal afferent inputs from the cochlea leading to a cascade of plastic changes. Animal studies indicate that deafferentation can result in over-representation in the cortical tonotopic map within primary field (A1) neurons of frequencies at the edge of the hearing loss, increased spontaneous activity likely due to reduced GABAergic inhibition and increased synchronous activity (interneuronal correlations) in the region of hearing loss (Adjamian et al. 2009; Eggermont and Roberts 2004; Middleton et al. 2011; Noreña 2010).

If tinnitus is the unfortunate perceptual consequence of neuroplastic events related to hearing loss, then interventions that promote changes in frequency representation, inhibitory activity or pathological synchronous firing at the level of the cortex, and potentially disrupt the tinnitus generating activity, are very much of interest. One intervention that has attracted interest in recent years is frequency discrimination training. In our recent systematic review of auditory perceptual training for tinnitus, we identified six studies evaluating the benefit of frequency discrimination training (Hoare et al. 2010). All these studies restricted training stimuli to single-frequency tones somewhere within the region of hearing loss—hypothesising that such stimulation might effectively ‘feed the deprived zones of the tonotopic map’. Although five of those studies reported statistically significant reductions in tinnitus intrusiveness (Flor et al. 2004; Herraiz et al. 2006, 2007, 2009, 2010), all had methodological weaknesses. We were particularly critical about the choice of appropriate control conditions, limited outcome measures of self-reported or psychoacoustic features of tinnitus (never both), lack of stable baseline measures before intervention and the limited evaluation of long-term retention. For example, while animal studies measure the psychoacoustic properties of tinnitus (dominant pitch and sensation level), the most desired outcome for people with tinnitus is a reduction in self-reported distress. Both types of outcome measure are therefore necessary to bridge the gap between animal and human literature. To date, only one study has examined the change in tinnitus percept after frequency discrimination training and in only one participant (Noreña et al. 2006). Measures of the tinnitus spectrum showed an almost complete extinction of high-frequency components (>8 kHz), after training at four frequencies ranging 3.3–6.5 kHz. Whether this spectral change could be associated with a clinically relevant measure of tinnitus (such as self-reported handicap) is still unknown.

The premise in previous studies of frequency discrimination training for people with tinnitus has been that it promotes some degree of reorganisation within primary auditory cortex, thereby disrupting the tinnitus-generating network. Links have been drawn with training in normal-hearing (non-tinnitus) animals. In particular, frequency discrimination training has been shown to increase the number of neurons tuned to the trained frequency (Recanzone et al. 1993). Active listening evidenced by *perceptual learning* appeared to be essential for tonotopic reorganisation to take place since animals who received the same stimulus in a passive listening context did not show such neural changes. Contrary to this, Noreña et al. (2006) reported tonotopic map reorganisation in juvenile cat auditory cortex as a result of long-term *passive* exposure to a spectrally enhanced acoustic environment, without any resulting hearing loss, so *learning* as a result of sound exposure does not appear essential for cortical reorganisation to occur due to the sound stimulus. Earlier, Noreña and Eggermont (2005) demonstrated that compensatory sound enrichment can also interrupt the typical reorganisation in A1 observed as a result of noise-induced hearing loss. The putative mechanisms of cortical reorganisation as a result of frequency discrimination training therefore may not require the participant to learn, but simply to be repeatedly and extensively exposed to appropriate sound signals.

Auditory perceptual training might equally be effective through strengthening or dissipating neuronal activity associated with other proposed mechanisms of tinnitus generation. For example, training might serve to increase or strengthen lateral inhibitory circuits that are depleted as a result of hearing loss. Indeed, lateral inhibition is the proposed mechanism of tinnitus benefit from passive forms of sound enrichment such as notched music therapy (Okamoto et al. 2010) and the low-rate electrical stimulation reported to extinguish tinnitus in a cochlear implantee reported by Zeng et al. (2011). The auditory cortex is rich in lateral inhibitory GABAergic circuits (Prieto et al. 1994). Middleton et al. (2011) demonstrated how a reduction in GABAergic inhibition results in hyperactivity in the dorsal cochlear nucleus in a mouse model of tinnitus. Recently, Diesch et al. (2010) presented human cortical data consistent with a role for reduced lateral inhibition in tinnitus. This was evidenced by reduced amplitude of the steady-state response to multiple component amplitude-modulated tones compared to single-component stimuli in the group of subjects with tinnitus, compared to control subjects who were matched for hearing loss but who did not experience tinnitus. This may represent a physiological marker for tinnitus and a potentially fruitful therapeutic target for sound-based interventions. Benefit might be derived from training at frequencies that do not correspond to the dominant tinnitus pitch itself, but at adjacent frequencies that drive neurons with inhibitory connections to them.

Another proposed mechanism of tinnitus generation involves enhanced interneuronal correlations whereby an ensemble of auditory neurons fire in a (pathologically) stabilised synchronous way. Such activity is typically observed in the primary auditory cortex in animal models of tinnitus where there has been an induced hearing loss (Engineer et al. 2011; Noreña and Eggermont 2003; Seki and Eggermont 2003). In humans, a role for synchronous activity in tinnitus is implicated by Weisz et al. (2007) who examined activity in left and right auditory cortices using MEG and found that oscillatory activity was greater in people with tinnitus than in controls and that it tracks laterality of the tinnitus percept (Weisz et al. 2007). Further supporting this position, van der Loo et al. (2009) reported an EEG study involving a cohort of subjects with unilateral tinnitus, showing that resting-state gamma band oscillations in the auditory cortex contralateral to the site of tinnitus had a significant positive correlation with self-reported tinnitus loudness rating. Again, there is reason to suggest that frequency discrimination training could modulate such synchronous activity. For example, repeated and persistent stimulation of a particular subset of neurons within a region of pathological synchronous activity could promote the asynchronous firing of that subset of neurons and terminally interrupt the synchronous pattern (Hauptmann and Tass 2007). In the case of tinnitus, this could presumably work either by directly forcing neurons within the tinnitus-generating region to fire out of synchrony with others within the ensemble, or by promoting asynchronous firing from outside the ensemble through a stochastic pattern of lateral inhibition.

So while there is good reason to investigate the potential of frequency discrimination training, there are many basic parameters that need to be systematically investigated. It is interesting to note for example that no human studies have compared the efficacy of training using sounds that stimulate normal versus hearing-impaired regions of A1 (Hoare et al. 2010). It therefore remains an open question as to which regime of sound-based training has the greatest influence on tinnitus outcome. A related uncertainty concerns the pitch relationship between the training regime and the dominant tinnitus pitch. Although sound enrichment in a high-frequency hearing loss region might be effective in driving plastic changes in A1, high-pitched sounds may be less comfortable for prolonged exposure and may therefore reduce compliance on the task. Hence, a second important comparison is to try to separate the contribution of tonotopic location of activity (spectral characteristics) from the pitch characteristics of the training stimulus. A third important question concerns the duration of training. Again, this has not been systematically addressed in any of the previous training studies that we reviewed, although a post hoc analysis conducted by Flor et al. (2004) indicates that those people who trained for longer reported more benefit.

Here, we address these three outstanding questions in two novel experiments that also go some way to resolve our criticisms of those earlier studies. Specifically, we make comparisons with a previously untested active control condition (training within the region of normal hearing), we adopt both self-report and psychoacoustical outcome measures of tinnitus, we provide full familiarisation of those measurement procedures before the baseline assessment and we obtain data at 1-month follow-up. In study 1, a double-blind randomised controlled design assessed the effect of frequency discrimination training using (A) a single frequency in the region of normal hearing, (B) a single frequency in the region of hearing loss, or (C) a high-pass (missing fundamental) harmonic-complex tone that provided high-frequency stimulation but evoked a low-pitch percept. Informed by the general trends reported in our systematic review (Hoare et al. 2010), it could be hypothesised that frequency discrimination training at frequencies corresponding to hearing loss (i.e. groups B and C) should improve self-reported tinnitus handicap and modify the tinnitus percept (bandwidth, dominant pitch or loudness). According to this hypothesis, group A was considered the active control condition, although given the alternative potential mechanisms of action for frequency discrimination training we might predict otherwise. In study 2, a single-blind randomised design assessed the influence of training period (intensity and duration) using (D) 15-min sessions five times a week for 4 weeks, and (E) 60-min sessions five times a week for 2 weeks. We hypothesised that the intensity of training would be positively associated with the magnitude of benefit.

## Methods

This work is reported according to the CONSORT statement for randomised trials of nonpharmacological treatments (Boutron et al. 2008).

### Participants

Participants were recruited through advertisement in local ear, nose, and throat and audiology departments and on our departmental website. Participants were adults with chronic subjective tinnitus (experienced for greater than 6 months) who had a ≥40 dB hearing loss on at least one test frequency (0.125, 0.5, 1, 1.5, 2, 3, 4, 6, 8, 9, 10, 11.25, 12.5 and 14 kHz) in at least one ear and were not currently receiving any therapy or other intervention that could affect their hearing or tinnitus. Participants with significant hyperacusis, anxiety or depression were excluded, as were participants with no substantial hearing loss (thresholds of <40 dB at all test frequencies) or hearing loss ≥40 dB at all test frequencies. All participants gave their written informed consent to take part in the study in accordance with the ethical approval granted by Derbyshire (UK) Research Ethics Committee.

### Audiometry

Pure-tone audiometry was conducted in a sound-proofed booth using the Siemens Unity 2 system and Sennheiser HDA 200 headphones. The steepness of hearing loss was determined according to whether there was ≥40 dB hearing loss per octave (considered steep-sloping hearing loss) or <40 dB hearing loss per octave (considered gradual sloping hearing loss) within the frequency range tested (125 Hz to 14 kHz).

### Frequency discrimination training procedure

Frequency discrimination training was delivered using STAR software (Barry et al. 2010). Participants were loaned a laptop computer with a Yoga AD-200 USB Adaptor soundcard and Sennheiser HD 25 headphones, so that all training could be performed at home using a carefully calibrated system. Training comprised sounds that were presented in a three-interval, three-alternate forced choice (3I-3AFC) ‘oddball’ paradigm. For a given individual, the ‘trained’ fundamental frequency (the standard) was fixed throughout, and the target (the oddball) differed by percentage (Hertz) above the fundamental frequency of the standard. An adaptive staircase procedure maintained oddball discrimination performance at 79 %. The duration, date, time and performance throughout the training session was automatically logged by the computer. The fundamental frequency discrimination threshold was calculated as the geometric mean of the last two reversals in each training session. Each training session consisted of one adaptive run. The ‘base’ sound level for training was fixed at 55 dB sensation level (SL) according to better ear threshold measured at the training frequency. Level was roved within trials by ±6 dB SPL to remove loudness cues (c.f. Thai-Van et al. 2002, 2003). Full training on the procedure was given in the laboratory before participants were given the laptop computer to take home. They were encouraged to contact the researcher issuing the laptop if there were any questions or problems over the training period.

In study 1, all participants (groups A, B and C) were instructed to perform the training task for 30 min, five times a week over a 4-week period (20 sessions in total). In study 2, participants were instructed to perform the task for 15 min, five times a week over a 4-week period (20 sessions in total, group D) or for 60 min, five times a week over a 2-week period (10 sessions in total, group E). Compliance with training was measured as time on task, as recorded by the training software.

### Training frequencies

**FIG. 1:**
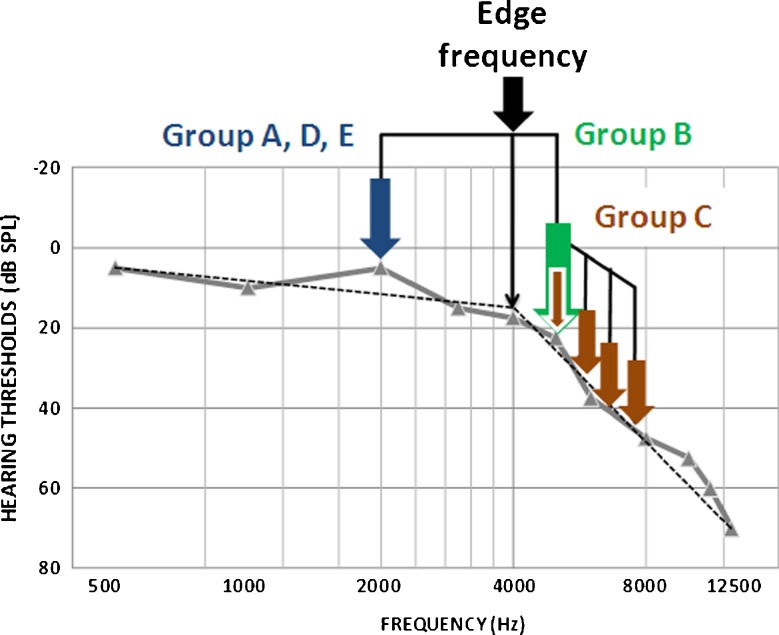
Training frequencies were defined according to the audiogram. Participants were randomly assigned to train at pure tone frequencies in their region of normal hearing (1 octave below the audiometric edge frequency), in their region of hearing loss (1/4 octave above the edge), or on harmonic sounds that span the region of hearing loss (beginning 1/4 octave above the edge frequency) which have a percept in the region of normal hearing. In this example, if the participant was allocated to one of groups A, D or E, they would train at 2 kHz. If they were allocated to group B, they would train at 5 kHz. If they were allocated to group C, they would train at a harmonic composed of 5, 6, 7 and 8 kHz tones, which would generate a low pitch (∼1 kHz).

Choice of training stimulus was individually tailored according to the audiometric edge frequency derived from the audiometric profile (125 Hz to 14 kHz; see Fig. 1). To compute the ‘edge’, a bespoke Matlab procedure was used to fit a ‘broken-stick’ function to the audiogram using a nonlinear regression with either one (H1) or two breaks (H2; c.f. Sereda et al. 2011). The best-fitting ‘broken-stick’ function was then selected using a parametric bootstrap approach. Participants in groups A, D and E were trained on a single-frequency standard within the normal-hearing range, defined as one octave below the audiometric edge. Participants in group B were trained on a single-frequency standard in the region of hearing loss, defined as a frequency that was at least 1/4 octaves above the audiometric edge, and at which hearing threshold was greater than 20 dB SPL. Participants in group C trained on a four-component harmonic complex (*n* kHz, *n* × 1.2, *n* × 1.4, and *n* × 1.6, where *n* was determined by the same rule as for group B). This was a high-pass, missing fundamental tone with a timbre in the high-frequency range, but a pitch corresponding to a low-frequency tone.

### Self-reported tinnitus handicap and other questionnaire measures

The Tinnitus Handicap Questionnaire (THQ; Kuk et al. 1990) was the primary measure of training efficacy. It is a validated measure of tinnitus severity and has a reported test–retest repeatability (6–8 weeks intertest interval) of 84 % (Henry and Wilson 1998). Crucially for the present studies, the THQ is proposed as a more sensitive measure of change than the more commonly used Tinnitus Handicap Inventory (Meikle et al. 2008). Twenty-seven questions provide a global measure of tinnitus handicap; the greater the score, the more severe the handicap (maximum score = 2,700). We used version 2 of the questionnaire (available at www.uihealthcare.com) in which questions 1 and 8 are reverse scored (100 minus participants score). THQ scores of >600 indicates tinnitus intrusiveness that disrupts daily activity (Sullivan et al. 1993). In addition, the THQ assesses three distinct factors: factor 1—physical health, emotional and social consequences of tinnitus (15 questions, maximum score = 1,500); factor 2—hearing difficulty (eight questions, maximum score = 800); and factor 3—the individuals’ outlook on tinnitus (four questions, maximum score = 400). Factor 3 however has low internal consistency and test–retest reliability (Kuk et al. 1990; Newman et al. 1995) and so is not reported here. Following our previous recommendations (Hoare et al. 2010), the THQ was administered twice before training. Results of the initial assessment are not reported and the second measure was taken as baseline (T0).

The initial assessment also included a case history, the Beck Anxiety Index and Beck Depression Index (BAI and BDI, respectively; Beck et al. 1997) and the Hyperacusis Questionnaire (Khalfa et al. 2002). Self-reported tinnitus loudness and tinnitus annoyance using a visual analogue scale (0–100) were also collected at baseline.

### Psychoacoustic properties of tinnitus

The ‘Tinnitus Tester’ (Roberts et al. 2008) was used to assess qualities of the tinnitus sensation (sensation level, tinnitus bandwidth and dominant tinnitus pitch) over a 0.5–12 kHz frequency range. Participants matched loudness by adjusting the level of a range of sound clips (centre frequencies, 0.5–12 kHz) until each one was perceived to equal that of the tinnitus sound. We took the loudness measure as the matched value at a single-frequency (typically at 0.5 or 1 kHz) corresponding to little or no hearing loss and distant from the dominant tinnitus pitch. SL was then calculated as an adjusted decibel value that took account of any hearing loss at that frequency. A profile of tinnitus spectrum representing bandwidth was generated by asking participants to rate the likeness of the same 11 sounds used for loudness matching (centre frequencies, 0.5–12 kHz) to the pitch of their tinnitus, using a 100-point scale. A unit of bandwidth was calculated as the standard deviation of all frequencies in the tinnitus spectrum, where each frequency was weighted by its percentage likeness to the tinnitus pitch identified by the participant (c.f. Sereda et al. 2011). The dominant tinnitus pitch was that frequency in the spectrum which had the highest likeness rating. Again, following our previous recommendations (Hoare et al. 2010), the Tinnitus Tester was administered twice before training. Results of the initial assessment are not reported and the second measure was taken as T0.

### Sample size

Sample size was estimated using data from studies of comparable duration, involving standard tinnitus therapies including passive sound stimulation (Henry et al. 2006; Londero et al. 2006), and which used the global THQ (range, 0–2,700) as their outcome measure. A two-sample *t* test power analysis (G*Power 3 software) was used to determine the number of participants required to show a significant difference between our main points of interest, i.e. the difference between baseline THQ score and the score at follow-up. For a two-sided significance level of 0.05 and 80 % power, 11 participants were estimated for each training group. Given the dropout of 20 % reported in Londero et al. (2006), our goal for recruitment was 14 participants per group.

### Allocation of participants to training groups

In study 1, participants were randomly allocated using a minimisation protocol (Altman and Bland 2005). In minimisation schemes, the next allocation depends on characteristics of those already allocated. Allocation of each participant group thereby ensures overall balance of prognostic factors between groups. The aim of minimization here was to generate groups that were evenly matched according to age, gender and whether their hearing loss slope was steep (≥40 dB per octave) or gradual (<40 dB per octave). Steep-sloped hearing loss has been associated with changes in the auditory cortex that correlate with tinnitus (König et al. 2006; Sereda et al. 2011). In study 2, participants were again randomly allocated using the minimisation protocol, matching according to age, gender and tinnitus severity (global THQ score <600, 600–1,200, >1,200).

A number of steps ensured blinding to group allocation. First, the minimisation was performed by a researcher who was otherwise not involved in the study. Second, all participants were unaware as to which training groups were expected to improve. In study 1, the researcher measuring training outcome was always blind to group allocation. In other words, the researcher who completed the pre-training assessment (T0) and instructed the participant on the training procedure was not the same person who assessed the participants after training (T1, T2, T3). In study 2, this was not possible as the time course for training and assessment for the two groups was different. However, participants were blind as to which group was expected to show most benefit.

### Data analysis

Analysis was conducted on an intention-to-treat basis such that once a participant commenced training, they were included in the final analysis. Where participants left the study before completion, missing values were imputed using appropriate methods. For THQ scores, missing data amounted to 4.9 % of the total. These missing values were imputed using an expectation–maximisation method which assumes a normal distribution for the partially missing data and bases inferences on the likelihood under that distribution (maximum 25 iterations, SPSS v16.0). Normal distribution of the data was confirmed by the Kolmogorov–Smirnov statistic (*p* = 0.200). For psychoacoustic measures of tinnitus sensation level, bandwidth and dominant pitch, the last-observation-carried-forward method was used. This method is more conservative but was considered sufficient and appropriate. The reasons were (1) the relative resistance of psychoacoustic measures to placebo effects (as cautioned in Streiner (2008)), (2) the known weak relationship between psychoacoustic outcomes and reported tinnitus benefit and (3) national guidelines advocating the main clinical utility of psychoacoustic measures for demonstrating to the patient stability over time (Department of Health 2009).

Main analyses were conducted using analysis of variance designs that included significant covariates (BDI score, BAI score, age, gender and slope of hearing loss, as appropriate) to model the influence of potential confounding factors. Where Mauchly’s test indicated that the assumption of sphericity had been violated, degrees of freedom were corrected using Greenhouse–Geisser estimates of sphericity.

As is appropriate for repeated-measures designs, clinical effect sizes were calculated as partial eta-squared (*η*_*p*_^2^) where *η*_*p*_^2^ > 0.02 = a small, *η*_*p*_^2^ > 0.09 = a moderate, and *η*_*p*_^2^ > 0.25 = a large effect size (Bakeman 2005).

## Results

**FIG. 2:**
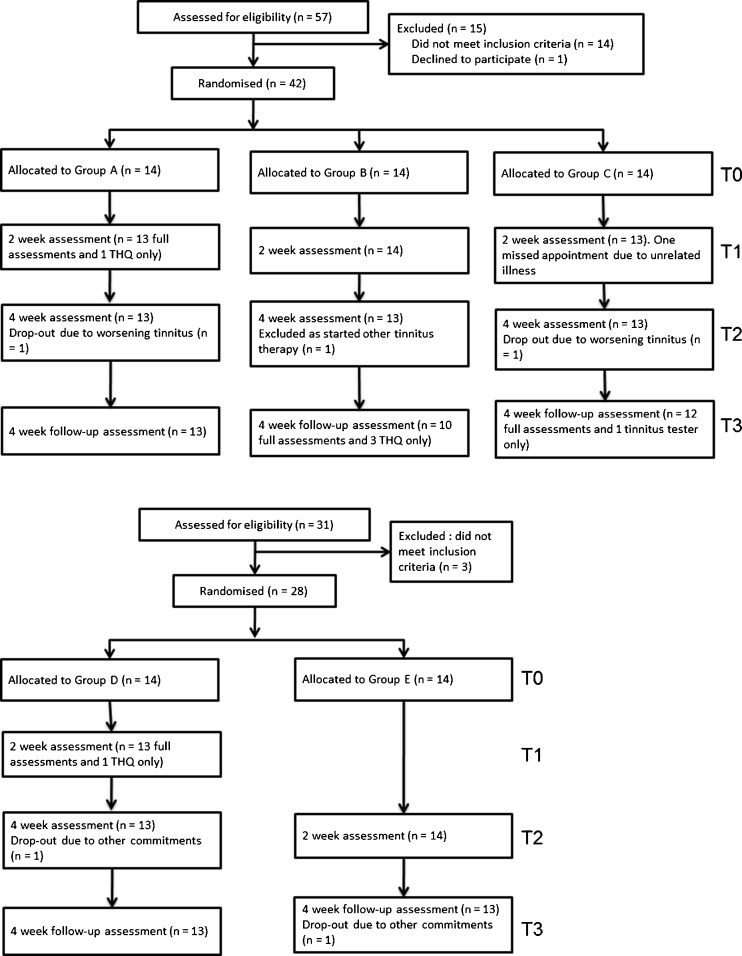
Flowchart of participants per group.

Recruitment began in April 2010 and follow-up assessments were completed in August 2011. Figure 2 reports the flow of participants through each study. Of 70 participants who met our inclusion criteria and commenced training, four left the study after completing approximately half of the required training; one each from groups A and C complained of worsening tinnitus and one each from groups D and E because of other commitments. One participant from group B was excluded at the post-training assessment as he started taking night sedation towards the end of his training period.

### Hearing thresholds and estimate of tinnitus spectrum

**FIG. 3:**
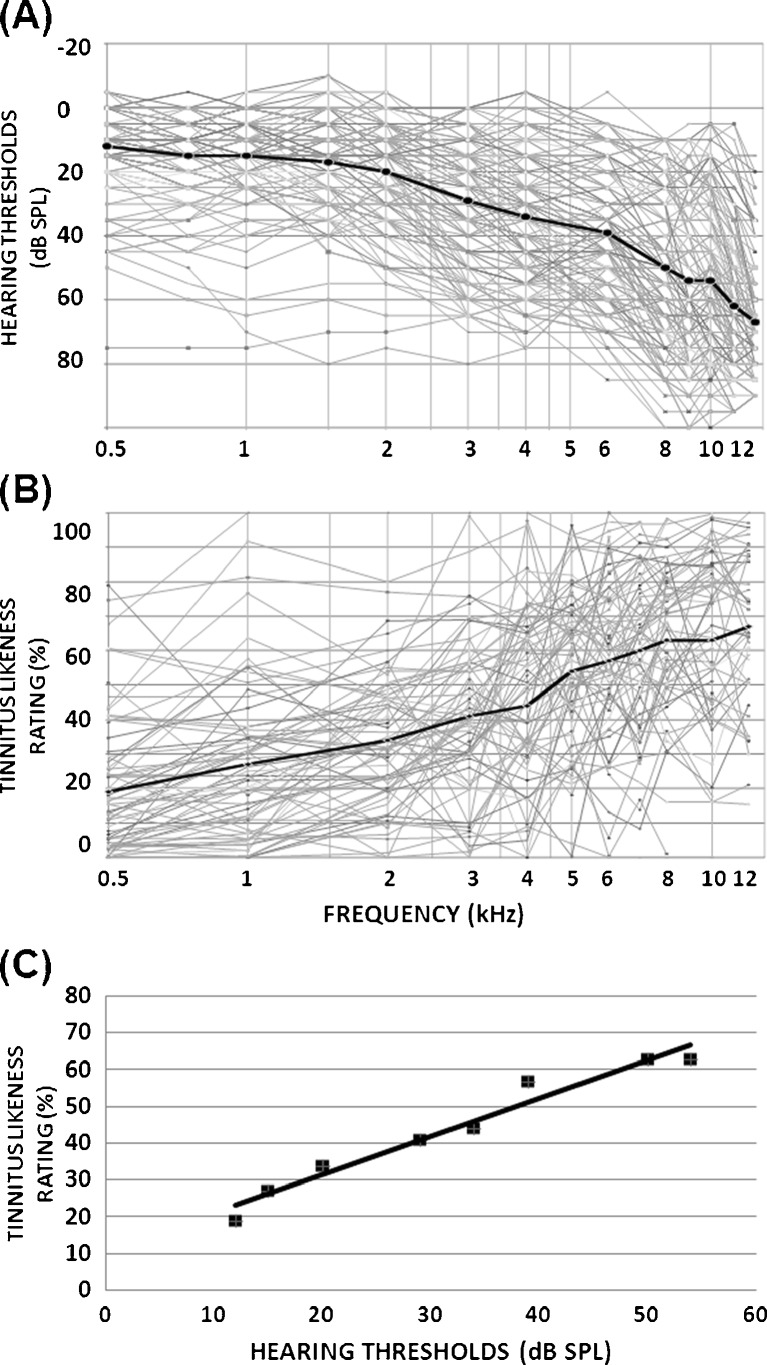
Hearing thresholds and tinnitus likeness. **A** Hearing loss (both ears) of all 70 participants are given as *light grey*, mean threshold is given in *black*. **B** Tinnitus likeness spectrographs for all 70 participants are given in *grey*, mean likeness ratings are given in *black*. **C** The graph illustrates a strong association between mean hearing threshold and mean tinnitus likeness of the same frequencies.

In both studies, groups were evenly matched for (0.5, 1, 2, 4 kHz) audiogram pure-tone average. For study 1, a one-way ANOVA showed no significant difference in pure-tone average across groups A, B and C [*F*(2, 38) = 0.234, MSE = 40.89, *p* = 0.793]. For study 2, an independent samples *t* test showed that there was no significant difference in pure-tone average between groups D and E [*t* (26) = 1.07, *p* = 0.295] (Table 1). Figure 3a illustrates the mean and individual audiograms (both ears) for all 70 participants. In general, hearing loss was moderate to severe at high frequencies (6 kHz and above). Figure 3b shows the baseline tinnitus spectra based on a likeness rating for all 70 participants, showing increasing reported contributions at higher frequencies. Tinnitus bandwidth was matched across groups. For study 1, a one-way ANOVA showed no main effect of group (A, B and C) on tinnitus bandwidth [*F*(2, 38) = 1.31, MSE = 541182.4, *p* = 0.281], and in study 2 also an independent samples *t* test confirmed that groups D and E did not differ from each other at baseline with respect to tinnitus bandwidth [*t* (26) = 0.031, *p* = 0.976]. There was a significant correlation between the degree of hearing loss at each test frequency and the contribution of that frequency to the tinnitus sound, i.e. the mean contribution of a frequency to the reported tinnitus sound was proportional to the mean degree of hearing loss at that frequency. This was true for the whole cohort of 70 participants (Fig. 3c, *r* = 0.988, *p* < 0.001) as well as within each of the five groups (*r* ≥ 0.926, *p* < 0.001 in all cases).

**TABLE 1:** Baseline characteristics and training details

Measure	Group A	Group B	Group C	Group D	Group E
	Mean (range)	Mean (range)	Mean (range)	Mean (range)	Mean (range)
Gender	10 M, 4 F	9 M, 5 F	11 M, 3 F	9 M, 5 F	9 M, 5 F
Age	57.5 (29–70)	55.7 (41–69)	54.4 (36–68)	56.3 (27–75)	59.6 (42–85)
Pure-tone average (dbHL)	17.6 (6–56)	19.2 (4–40)	15.8 (0–59)	20.2 (6.3–37.5)	16.4 (5–32.5)
Hearing loss slope	8 Steep, 6 gradual	6 Steep, 8 gradual	7 Steep, 7 gradual	8 Steep, 6 gradual	10 Steep, 4 gradual
Tinnitus duration (years)	13.7 (0.66–50)	9.4 (0.5–20)	16.9 (2–51)	9.8 (1–30)	9.5 (0.5–27)
BDI	2 (0–10)	0.5 (0–1)	2.6 (0–9)	2.4 (0–7)	3.4 (0–13)
BAI	7.6 (1–15)	3.7 (0–8)	9.1 (0–21)	5.6 (0–14)	10.4 (4–24)
Hyperacusis	12.1 (1–18)	10.4 (2–22)	15.9 (5–27)	15.1 (1–28)	13.3 (6–20)
Global THQ	1,054 (568–1,746)	760 (423–1,238)	1,165 (637–1,640)	1,255 (568–2,100)	1,155 (231–2,030)
Subscale 1	37.4 (10–67.1)	22.5 (3–40.6)	37.7 (18.5–59.3)	43.1 (10.8–84.7)	40.4 (2.1–88.7)
Subscale 2	34.1 (9–54.9)	30.2 (6.3–73.5)	47.5 (16.5–71.3)	50 (15.8–70)	39.8 (0.3–79.3)
Self-reported annoyance (/100)	30.9 (0–70)	28.2 (1–100)	29.5 (0–70)	36 (0–100)	40 (0–100)
Self-reported loudness (/100)	47.4 (20–100)	39 (21–80)	44.6 (30–75)	47.1 (21–81)	52.1 (21–93)
Sensation level (dbSL)	21.6 (4–68)	16.6 (2–26)	25.4 (8–57)	24.4 (5–52)	19.1 (1–41)
Dominant Tinnitus pitch (kHz)	9.9 (0.5–12)	8.1 (0.5–12)	7.4 (7–12)	7.6 (2–12)	7.4 (3–12)
Tinnitus bandwidth (units)	3.3 (2.3–3.8)	2.9 (0.6–3.7)	3.2 (1.8–3.7)	3.3 (1.9–4)	3.3 (2.5–3.9)
Training frequency (kHz)	1.8 (0.25–4)	3 (1.5–6)	5 (1.5–10)	1.1 (0.5–4)	1.1 (0.5–2)

### Other baseline characteristics

Table 1 shows mean baseline characteristics of each group. In study 1 (Groups A, B and C), one-way ANOVA revealed no significant between-group differences in baseline for duration of tinnitus, self-reported tinnitus annoyance, dominant tinnitus pitch, self-reported tinnitus loudness or psychophysical match of tinnitus sensation level (*p* > 0.05). A one-way ANOVA used to assess baseline global THQ score of the three groups (A, B or C) was however significant [*F*(2, 38) = 5.533, MSE = 612531.5, *p* = 0.008]. A post hoc Tukey’s HSD test showed that the mean baseline THQ score for group B was significantly lower than that of group C (*p* < 0.05). All three groups however had a mean baseline tinnitus handicap sufficient to disrupt daily activity (i.e. >600; Sullivan et al. 1993).

In study 2, participants were minimised according to baseline global THQ scores. Hence, there was no significant difference in baseline global THQ scores between groups D and E [*t* (26) = 0.474, *p* = 0.639]. Once again, in study 2 there were no significant between group differences in baseline duration of tinnitus, self-reported tinnitus annoyance, dominant tinnitus pitch, self-reported tinnitus loudness or psychophysical match of tinnitus sensation level, or bandwidth of the tinnitus spectrum (*p* > 0.05).

### Training

Compliance with the training regime was equivalent across all five groups [*F*(4, 64) = 2.125, MSE = 506.679, *p* = 0.087]. In study 1, compliance with prescribed training time (10 h over a 4-week period for all participants) was high: 94 % in group A, 92 % in group B and 95 % in group C. In study 2, compliance was equally high for group D (95 %) which trained for 5 h over 4 weeks, but slightly lower (81 %) in group E which trained for 10 h over 2 weeks.

We measured perceptual learning on the training task as a potential correlate with changes in tinnitus percept. An omnibus test was conducted to include all five groups, the within-subject factor of training session (T0, T2), and the between-subject factors of training stimulus and duration. Across all participants, there was a significant main effect of training session [*F*(1,61) = 5.23, MSE = 998307.452, *p* < 0.05], i.e. there was a significant overall reduction in discrimination threshold after training. Between-subjects analyses showed that there was also a significant main effect of training stimulus [*F*(2,61) = 10.343, MSE = 4738659.77, *p* < 0.001]. Post hoc comparisons showed that the reduction in discrimination threshold (perceptual learning) observed for participants who trained on the harmonic complex (group C) was significantly smaller than the reductions observed among those trained using pure tones (*p* < 0.01). In fact, for four participants in group C, the discrimination threshold was the same or *increased* in the last compared to the first training sessions. Only one participant in groups A and D, and two participants in group E did not show a reduced discrimination threshold after training. All participants in group B showed a reduction in discrimination threshold after training.

There was no main effect of training duration (5 versus 10 h) on perceptual learning [*F*(2,61) = 0.025, MSE = 11594.56, *p* = 0.975], suggesting as might be expected, that much of the total perceptual learning on the training task occurred in the first few hours of training. No significant interactions between training session and training stimulus or duration were found.

### Tinnitus handicap

#### Omnibus analysis on global THQ scores

**FIG. 4:**
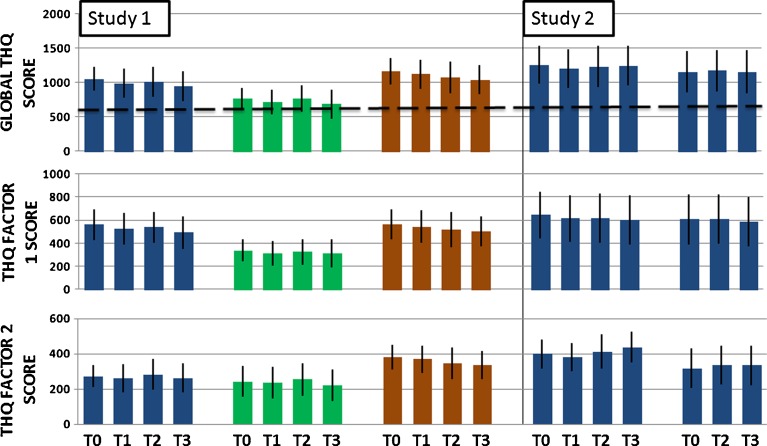
Global and THQ factor scores. Mean THQ scores (±95 % CI) per group at baseline (T0), after 2 weeks training (T1), after 4 weeks training (T2), and at a 1 month follow-up. *Broken line* cut-off score for bothersome tinnitus. Subsets of questions give THQ factors 1 (emotional component) and factor 2 (effect on hearing) scores. T0 is the second pre-training assessment, which is taken as the stable baseline. T1 is the mid-training assessment, T2 is post-training, and T3 is a 1 month follow-up assessment; *n* = 14, per group.

Mean THQ scores from both studies are given in Figure 4 (data is also provided as Electronic supplementary material (ESM) 1). To assess the three main factors in studies 1 and 2, a single well-powered analysis was conducted which pooled THQ data from all 70 participants. A mixed-design ANOVA was used to assess (1) the within-subject effects of training session (THQ scores at T0, T1, T2 and T3), (2) the between-subjects effects of training stimulus (pure-tone low frequency (groups A, D E), pure-tone high frequency (group B), harmonic complex (group C)) and of training duration (20 × 30 min for 4 weeks; groups A, B and C), 20 × 15 min for 4 weeks (group D) and 10 × 60 min for 2 weeks (group E), and (3) the significance of any two-way interactions between training session and training stimulus, or training session and training duration. Only BDI score emerged as a significant covariate with global THQ and so only this variable was included as a covariate.

For clarity, the outputs from ANOVA tests are given in Table 2. Within-subjects tests revealed a statistically significant, but clinically small effect: global THQ score decreased over time, with a small effect size, *η*_*p*_^2^ = 0.059. Out of the 14 participants in each group, similar numbers reported reduced Global THQ at T3 compared to T0 (group A = 9, group B = 9, group C = 10, group D = 6, group E = 7). Although overall, training reduced reported tinnitus handicap, no pair-wise comparison between T0, T1, T2 and T3 was statistically significant (*p* > 0.05). Choice of stimulus training had no influence on reported handicap. Neither was there a main effect of duration of training on THQ scores overall. There was no interaction between training session and stimulus or between training session and duration. Results for our primary outcome measure therefore suggest that there is a more generalised benefit of training rather than benefit that is specific to a particular stimulus or regime of training, and that it has a small clinical effect.

**TABLE 2:** Output from mixed-design ANOVA assessing the main effects and interactions of training session, stimulus and duration on Tinnitus Handicap Questionnaire scores

Effect	ANOVA
Global THQ	
Training session	*F*(2.462,157.598) = 3.985, MSE = 69829.701, *p* < 0.05, *η*_*p*_^2^ = 0.059
Stimulus	*F*(2,64) = 1.1, MSE = 798034.938, *p* = 0.339, *η*_*p*_^2^ = 0.033
Duration	*F*(2,64) = 0.812, MSE = 588917.991, *p* = 0.449, *η*_*p*_^2^ = 0.025
Training session × stimulus	*F*(4.925,157.598) = 0.773, MSE = 13547.193, *p* = 0.569, *η*_*p*_^2^ = 0.024
Training session × duration	*F*(4.925,157.598) = 0.354, MSE = 6200.08, *p* = 0.877, *η*_*p*_^2^ = 0.011
THQ factor 1	
Training session	*F*(2.589,165.715) = 3.354, MSE = 94.983, *p* < 0.05, *η*_*p*_^2^ = 0.051
Stimulus	*F*(2,64) = 0.941, MSE = 1434.859, *p* = 0.395, η_p_^2^ = 0.029
Duration	*F*(2,64) = 0.256, MSE = 390.037, *p* = 0.775, η_p_^2^ = 0.008
Training session × stimulus	*F*(5.179,165.714) = 0.422, MSE = 11.63, *p* = 0.864, *η*_*p*_^2^ = 0.013
Training session × duration	*F*(5.179,165.714) = 0.28, MSE = 7.726, *p* = 0.928, *η*_*p*_^2^ = 0.009
THQ factor 2	
Training session	*F*(2.409,154.202) = 0.758, MSE = 58.743, *p* = 0.493, *η*_*p*_^2^ = 0.012
Stimulus	*F*(2,64) = 1.315, MSE = 2096.399, *p* = 0.276, *η*_*p*_^2^ = 0.039
Duration	*F*(2,64) = 2.567, MSE = 4092.747, *p* = 0.085, *η*_*p*_^2^ = 0.074
Training session × stimulus	*F*(4.819,154.202) = 0.904, MSE = 70.06, *p* = 0.478, *η*_*p*_^2^ = 0.027
Training session × duration	*F*(4.819,154.202) = 0.608, MSE = 47.155, *p* = 0.688, *η*_*p*_^2^ = 0.019
THQ factor 3	
Training session	*F*(2.635,168.63) = 2.264, MSE = 133.398, *p* = 0.091, *η*_*p*_^2^ = 0.034
Stimulus	*F*(2,64) = 0.616, MSE = 305.095, *p* = 0.543, *η*_*p*_^2^ = 0.019
Duration	*F*(2,64) = 0.312, MSE = 154.308, *p* = 0.733, *η*_*p*_^2^ = 0.01
Training session × stimulus	*F*(5.27,168.63) = 3.11, MSE = 183.265, *p* < 0.01, *η*_*p*_^2^ = 0.089
Training session × Duration	*F*(5.27,168.63) = 3.188, MSE = 187.88, *p* < 0.01, *η*_*p*_^2^ = 0.091

Partial-eta squared (*η*_*p*_^2^) of 0.02–0.09 is considered a small effect size

#### Secondary analyses

Patterns of change in THQ factor 1 scores (questions related to the emotional consequences of tinnitus) were comparable with the changes observed in global THQ scores. Change was assessed statistically using the same mixed-design ANOVA as for global THQ score. Across all 70 participants, there was a small effect of training session: THQ factor 1 score reduced over time (Fig. 4, ESM 1; Table 2) with a small effect size, *η*_*p*_^2^ = 0.051. Post hoc comparisons however revealed no pair-wise differences in THQ factor 1 score across T0, T1, T2 and T3 (*p* > 0.05). Again, neither training stimulus nor training duration influenced outcome on factor 1. There were no significant interactions of training session and stimulus or of training session and different training durations. As observed for global THQ score therefore, training resulted in a general improvement in the emotional consequences of tinnitus, rather than an effect specifically associated with a particular training stimulus or regime.

Changes in THQ factor 2 scores (questions related to the effects of tinnitus on hearing) were again assessed using the same mixed-design ANOVA as for the global THQ score. None of our manipulations affected factor 2 scores. Across all 70 participants, there was no statistically significant main effect of training session (Fig. 4, ESM 1; Table 2). Training therefore appeared to have no effect on the more functional aspects of tinnitus measured by the THQ.

#### Further post hoc observations

Global THQ was further analysed to determine the number of people reporting a categorical shift in their reported handicap. This was defined as a change in global THQ that represented a shift from intrusive tinnitus (>600 points on global THQ score) to non-intrusive tinnitus (<600 points on THQ score). At baseline, 62 out of our 70 participants had a global THQ score greater than 600 points, i.e. intrusive tinnitus. Of those, seven (11 %) showed an improvement at follow-up assessment that brought their score to below 600 points. Two were in each of groups A, B and C and one person was in group D. We examined whether there were any distinguishing features of these seven ‘improvers’ compared to the 55 ‘non-improvers’. The group of improvers had no obvious defining characteristics with respect to gender (four males, three females), slope of hearing loss (four gradual sloping, three steep-sloping) or age (range, 29–64 years). Strikingly though, whereas the average baseline Global THQ score for the non-improvers’ was 1,162 points (out of 2,700), the average baseline score for the seven improvers was significantly smaller at just 794 (*p* < 0.001, Fisher’s exact test). The effect of ‘improvement’ was not simply related to closeness to the 600 point score, however; whereas the improvers’ global THQ score reduced by an average of 383 points (48 %), non-improvers showed an average reduction of just 23 points (<2 %). This shows that those with a lower baseline THQ score to begin with were more responsive to this experimental intervention (frequency discrimination training), and suggests that those with a more intrusive tinnitus are more intractable.

Correlations analyses were conducted to look for significant relationships between training and changes in global THQ score. There was no significant association between changes in global THQ score and the individual selection of training frequency (*r* = −0.076, *p* = 0.633). Nor was there a significant association between the frequency separation of the training frequency and dominant tinnitus pitch and the change in THQ score (*r* = −0.122, *p* = 0.442). Overall, changes in global THQ score did not correlate with improvement on the training task (*r* = 0.206, *p* = 0.204). This implies that, rather than a stimulus-specific mechanism of improvement, more general mechanisms such as cognitive changes may be responsible for the improvements in THQ score in our participants.

Finally, pre-defined sub-analyses were conducted to explore the relationship between those characteristics used in the minimisation process and improvements in THQ score after training, i.e. are these factors predictive of who would improve in future studies? With respect to gender, 64 % of females improved, by an average of 102 global THQ points, whereas 56 % of males improved but by an average of only 53 global THQ points. This difference between males and females was not significant (*p* = 0.61, Fisher’s exact test). Of the 31 participants with gradual sloping hearing loss, 71 % showed an improvement by an average of 91 points on global THQ. Of the 39 participants with a steeply sloping hearing loss, only 49 % showed an improvement and by an average of 51 THQ points. This approached significance (*p* = 0.088 Fisher’s exact test). The final category, age, did not correlate with improvements in THQ score (*r* = 0.113, *p* = 0.35).

### Psychoacoustic characteristics of tinnitus

**FIG. 5:**
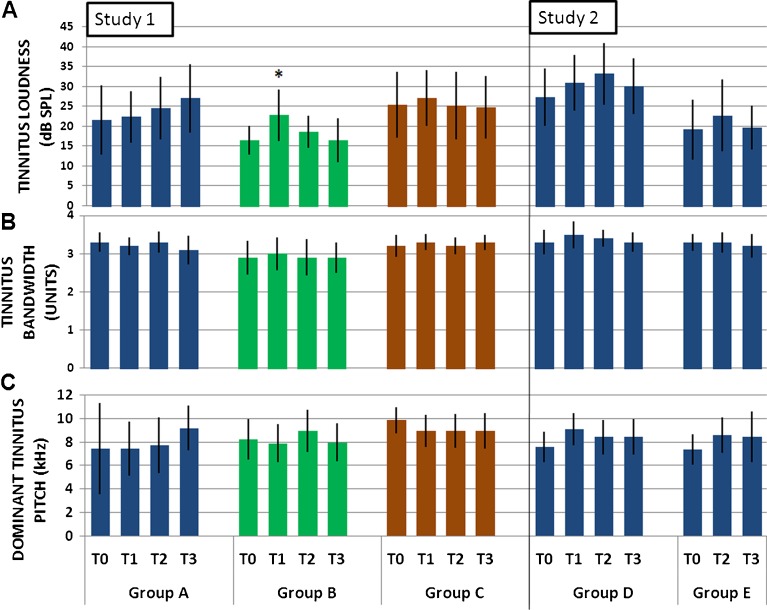
Tinnitus sensation level, bandwidth and dominant pitch. Mean measurements (±95 % CI) of **A** matched tinnitus sensation level, **B** tinnitus bandwidth and **C** dominant tinnitus pitch, in all groups. *p < 0.05, paired *t* test (corrected for multiple comparisons); *n* = 14, per group.

Baseline psychoacoustic characteristics of tinnitus are given in Table 1. Figure 5 gives mean values of each tinnitus characteristic per group across the four time points of the study.

#### Omnibus analysis of tinnitus sensation level

Tinnitus sensation level was matched to a hearing frequency that was not related to the dominant tinnitus pitch, typically at 0.5 or 1 kHz where individuals had hearing thresholds ∼0 dB SPL. Mean baseline tinnitus sensation level across all five groups was 21 dB SL (range was 1–68 dB SL, Table 1). Mean tinnitus sensation level at each assessment stage is given in Figure 5a.

As with our THQ data, sensation level data from studies 1 and 2 were analysed in a single mixed-design ANOVA to assess (1) the within-subject effects of training session (sensation level at T0, T1, T2 and T3), (2) the between-subjects effects of training stimulus (pure-tone low frequency (groups A, D and E), pure-tone high frequency (group B), harmonic complex (group C)) and of training duration (20 × 30 min for 4 weeks (groups A, B and C), 20 × 15 min for 4 weeks (group D) and 10 × 60 min for 2 weeks (group E)) and (3) the significance of any two-way interactions between training session and training stimulus, and training session and training duration. There were no significant covariates. All ANOVA statistics are reported in Table 3.

**TABLE 3:** Output from mixed-design ANOVA assessing the main effects and interactions of training session, stimulus and duration on tinnitus loudness, bandwidth and dominant pitch

Effect	ANOVA
Loudness	
Training session	*F*(3, 195) = 3.454, MSE = 162.642, *p* < 0.05
Stimulus	*F*(2,65) = 1.199, MSE = 760.792, *p* = 0.308
Duration	*F*(2,65) = 0.963, MSE = 611.149, *p* = 0.387
Training session × stimulus	*F*(6,195) = 1.677, MSE = 80.427, *p* = 0.128
Training session × duration	*F*(6,195) = 0.868, MSE = 41.625, *p* = 0.512
Bandwidth	
Training session	*F*(2.484,161.449) = 0.462, MSE = 78559.832, *p* = 0.673
Stimulus	*F*(2,65) = 1.692, MSE = 1923664.185, *p* = 0.192
Duration	*F*(2,65) = 0.465, MSE = 529131.5, *p* = 0.63
Training session × stimulus	*F*(4.968,161.449) = 0.594, MSE = 101021.807, *p* = 0.704
Training session × duration	*F*(4.968,161.449) = 0.38, MSE = 64698.902, *p* = 0.861
Dominant pitch	
Training session	*F*(3,189) = 0.522, MSE = 1.8, *p* = 0.668
Stimulus	*F*(2,63) = 0.552, MSE = 14.791, *p* = 0.579
Duration	*F*(2,63) = 0.25, MSE = 6.714, *p* = 0.779
Training session × stimulus	*F*(6,189) = 1.985, MSE = 6.843, *p* = 0.07
Training session × duration	*F*(6,189) = 1.346, MSE = 4.64, *p* = 0.239

Within-subjects tests revealed a significant main effect of training session on sensation level. Pair-wise comparisons revealed that tinnitus sensation level at T1 was significantly different to that at T0, at the 0.05 level of significance. Tinnitus sensation level increased significantly from 22 dB SL at T0 to 25.2 dB SL at T1 (Fig. 5a and Table 3). All other pair-wise comparisons were not significant. Mean tinnitus sensation level was reduced to 24.8 at T2 and 23.6 at T3. Between-subject analyses showed that there was no main effect of training duration or training stimulus on tinnitus sensation level and no interactions

#### Omnibus analysis of tinnitus bandwidth

Tinnitus bandwidth varied considerably between participants at baseline, from extremely narrow-band low-frequency sounds of 0.6 units wide, to broadband noise of 3.8 units (Table 1). Mean tinnitus bandwidth of each group at each time point is given in Figure 5b. Measures of tinnitus bandwidth were analysed using the same mixed-design ANOVA as described above for tinnitus sensation level. Within-subjects tests revealed no significant effects for any of the main factors or interactions (Table 3). Thus, training had no significant effect on tinnitus bandwidth.

#### Omnibus analysis of dominant tinnitus pitch

The average dominant tinnitus pitch of all 70 participants was 8 kHz at baseline (range, 0.5–12 kHz; Table 1). Dominant tinnitus pitch of each group at each time point is given in Figure 5c. Notably, groups trained at lower frequency (groups A, D and E) reported mean increases in dominant tinnitus pitch by T3, whereas both groups trained at hearing loss frequencies (B and C) reported a mean decrease in dominant tinnitus pitch at T3. The biggest mean change in dominant tinnitus pitch was observed in group A which reported an increase of 1.8 kHz (Fig. 5c).

Changes in dominant tinnitus pitch were assessed with the same mixed-design ANOVA as above, with age and slope of hearing loss included as significant covariates. Again, within-subjects tests revealed no main effect or interactions (Table 3). Between-subjects analyses showed that there was no main effect of training stimulus. Hence, again a mixed-design ANOVA revealed that training had no consistent effect on tinnitus pitch.

## Discussion

We first conducted a double-blind, randomised trial to assess the effects of frequency discrimination training on tinnitus percept and intrusiveness using (1) pure tones that were in the region of the participants normal hearing, (2) pure tones that were in their region of hearing loss or (3) harmonic sounds that span the region of hearing loss. We observed most change in both THQ scores and psychoacoustic measures of tinnitus in a group trained at normal-hearing frequencies. In our second study therefore, all participants were trained at normal-hearing frequencies, doing either less training (group D) or more intensive training over a shorter time-course (group E). Taking both studies together, there was a clinically meaningful and a statistically significant overall effect (Table 2); global THQ score reduced by an average of 76 points (7 %). However, against our starting hypothesis and the conclusions of previous studies, benefit was not specifically associated with training that provides enrichment at hearing loss frequencies. Rather, the number of participants reporting improvements in global THQ scores was roughly even across all five groups. We could conclude therefore that training at *any* frequencies equally results in some generic cognitive improvement such as changes in attention that has benefit for tinnitus in some individuals.

### Perceptual learning

Recanzone et al. (1993) demonstrated how learning on a frequency discrimination training task resulted in tonotopic change in A1, and that passive sound enrichment with the same stimuli did not have this effect. Here, we saw less perceptual learning in group C, the group trained on harmonic sounds, suggesting that this training was more difficult than training with pure tones. In normal-hearing listeners, Hall and Plack (2009) previously reported a ∼5-fold difference in the discrimination thresholds for a high-pass filtered harmonic sound with a pitch of 200 and a 200 Hz pure tone, supporting our observation here that pitch discrimination is challenging for missing fundamental harmonic-complex tones, perhaps due to a weaker pitch salience. However, despite the lack of significant perceptual learning in group C, there was as much benefit reported as in other groups, again suggesting that mechanisms that are not necessarily dependent on perceptual learning may be at play.

### Change in intrusiveness

All previous studies of frequency discrimination training for tinnitus benefit have focused on providing sound enrichment at hearing-loss frequencies to expand the representation of those frequencies in the auditory cortex, and have differentiated between the benefit of training at sounds matched to, or different from, the tinnitus pitch (Flor et al. 2004; Herraiz et al. 2006, 2007, 2009, 2010). All provided training at frequencies of 4 kHz or above and reported benefits in terms of a self-report or a change in validated questionnaire score. There was however a number of methodological issues with these studies which limit our confidence in their estimated effects (Hoare et al. 2010). Here, using a training regime comparable with previous studies, we established a stable pre-training baseline, and conducted our studies as single- or double-blind as was possible. Although there was an overall effect of training across all 70 participants, the lack of specific benefit for groups trained at hearing-loss frequencies is contrary to the conclusions of previous reports. The improvement observed in study 1 group A was not repeated in study 2 which involved the same training frequencies but with less prescribed training (group D) or less compliance (group E). One possible explanation that cannot be ruled out by the current data is that participants in group A did just enough (the minimum) training that is required to start to show benefit, and so training over a more prolonged period on the same task may produce more substantial benefit. Further studies involving training delivered over a number of months are required to fully understand the potential efficacy of this intervention.

It would also be interesting to further explore the observation that participants with a lower baseline THQ score are more likely to benefit from frequency discrimination training. Of the 62 individuals who started out with an intrusive tinnitus (>600), only seven reached a score below 600 (non-intrusive tinnitus) at follow-up assessment, while the remainder showed little reduction in global THQ (23 points out of 2,700). Frequency discrimination training therefore may only be indicated for people who have a mildly intrusive tinnitus to begin with, but for those individuals it may be an effective form of self-management. It seems less likely that training on its own would be an effective intervention for people with very intrusive tinnitus, but it may be a useful adjunct to other interventions such as education and counselling.

### Changes in tinnitus percept

Some changes in the perceptual characteristics of tinnitus related to training were observed. Notably, the early stages of training resulted in a temporary increase in tinnitus sensation level. This effect may be related to the need to attend to auditory stimuli during discrimination training, as hypothesised by Flor et al. (2004) who recorded a temporary increase in self-reported tinnitus severity after 1 week of frequency discrimination training.

Only a single case study has previously examined the effect of frequency discrimination training on the psychoacoustic characteristics of tinnitus. Noreña et al. (2002) trained one ear in one participant on four frequencies between 3.2 and 6.5 kHz and compared the tinnitus spectra before and after training in the trained and untrained ear. The participant reported a significant reduction in the high-frequency components of their tinnitus in the trained ear, resulting in a narrower tinnitus bandwidth. There was no significant change in tinnitus spectrum in the untrained ear, implying an effect of training. Tinnitus distress was not measured in this study however. The only other evidence for an effect of stimulus on the perceptual characteristics of tinnitus comes from studies of passive stimulation with hearing aids or tinnitus masking devices. For example, Moffatt et al. (2009) reported a reduction in the low-frequency components of tinnitus after low-moderate frequency hearing aid amplification. In another study, Schaette et al. (2010) found that, after 6 months of hearing aid or masker use, there was a reduction in tinnitus loudness and distress only in subjects with a tinnitus pitch of less than 6 kHz (i.e. within the amplification or output range of their device), suggesting that, as we predicted from our data, there may be a relationship between stimulus, tinnitus pitch and benefit.

Relating the changes we observed here to evidence from animal studies is difficult. Typically, the behavioural phenotype used in animal studies is an ‘all-or-nothing’ whereby the animal responds or does not respond in a training paradigm, interpreted as ‘the animal has or does not have tinnitus’. Our result has implications for those studies however. Although we did not report extinction of tinnitus in any case, the dominant tinnitus pitch changed by up to an average of 1.8 kHz within a single group. Such a shift in tinnitus sound in the animal studies mentioned here could result in a misinterpretation of tinnitus extinction, whereas in reality, it has simply shifted frequency.

### Mechanisms of change

We hypothesised that perceptual learning would be required for tinnitus benefit to be observed, yet we observed no differences in the change in tinnitus intrusiveness between group C which did not show significant perceptual learning, and other groups that did. In the literature, it has been shown that tonotopic change (which according to the reorganisational model is believed to be the plastic response required for disrupting the tinnitus-generating network) occurs in animals when perceptual learning on auditory discrimination tasks is observed. Other mechanisms of change may be more beneficial.

Herraiz et al. (2010) gave the first indication that training at tones that differed from the dominant tinnitus pitch has benefit above training at tones that have a frequency similar to or the same as the tinnitus pitch. This suggests that lateral inhibitory networks may be important here: stimulating specific frequency regions within A1 in ranges close to but not within the tinnitus frequency region would likely promote or strengthen lateral inhibitory activity (and perhaps thereby disrupt pathological synchronous activity) that would include to some degree the tinnitus generating region. As we show here, tinnitus bandwidth can be broad and generally mirrors the hearing loss region (Fig. 2). According to this view, stimuli delivered at normal frequencies might seem intuitively to be an effective method for promoting lateral inhibition.

As mentioned earlier, Flor et al. (2004) hypothesised that the need to attend to a stimulus that is similar to tinnitus may worsen its severity at the beginning of training. We similarly reported a significant increase in tinnitus loudness after an initial period of auditory training, which returned to baseline after training. This effect was most notable for group B which trained at frequencies most similar to the tinnitus sound of most individuals.

Finally, the potential that participants reporting benefit derived such from a general therapeutic relationship with researchers cannot be ignored. We found overall improvements in THQ factor 1 scores (improvements in emotional wellbeing), but no significant changes in factor 2 scores which addresses more functional aspects of tinnitus intrusiveness on hearing. Previous work has shown that simple information giving can be sufficient for some tinnitus patients to report improvements in tinnitus severity or handicap (Malouff et al. 2010). The presence of pronounced placebo effects in studies of tinnitus therapies is also notably high (Duckert and Rees 1984). Our study design controlled strongly for these effects, baseline measurements were repeated before intervention, studies were conducted blind, all participants received exactly the same information about the rationale for the study, and all participants then received an active intervention. Importantly, we measured psychoacoustical characteristics at every assessment, which provided the participant with a context to their tinnitus sound which for many was a stable repeated measure over time. This likely prevented any major placebo effect in self-reported intrusiveness, and for the more functional aspects of self report measured by the THQ in particular.

#### Future directions and conclusion

This systematic evaluation of frequency discrimination training has addressed many methodological issues observed in previous studies that have limited our confidence in the estimates of its effect on tinnitus. We too report a significant change in tinnitus intrusiveness after training but it is not specific to any particular stimulus, as previously supposed. Our findings also have implications for the interpretation of animal data as we observed shifts in dominant tinnitus pitch after frequency discrimination training that would be missed in animal behavioural work.

There is a need for continued evidence-based research to establish the efficacy of auditory training as a strategy for managing tinnitus. The many parameters that might be adjusted to optimise the benefits of auditory training include the number of trials (which may differ for different stimuli on the same task), or different stimuli duration, as suggested in studies of normal-hearing listeners (Roth et al. 2005, Wright and Sabin 2007). Combining auditory training with a second intervention may also be of benefit. In a recent study, Wright et al. (2010) demonstrated enhanced learning on a frequency discrimination task when training was supplemented with select periods of additional off-task stimulation with the same frequencies (this might be compared with a passive listening task not related to the training frequency such as listening to the radio). As the authors suggest, this could represent a mechanism for reducing the effort required to gain maximally from an auditory training regime, and so could be applied to frequency discrimination training for tinnitus. This approach would also support longer-term studies of auditory training for tinnitus. There is some suggestion in the data presented here that training over longer periods may lead to greater change in both perceptual characteristics and the intrusiveness of tinnitus. Longer-term follow-up is also desirable to shed light on whether the benefits reported here are indeed sustained beyond the 1 month follow-up assessment we performed.

Mechanisms of auditory learning include enhancement of both top-down cognitive processing and bottom-up sensory processing (for a review see Moore and Amitay 2007). Given the evidence for limbic system involvement in the emotional reaction to tinnitus (Lockwood et al. 1998, Mirz et al. 2000), it may be that more pro-active forms of training have benefit in terms of reward, and so the implementation of training that uses the principles of frequency discrimination training but which is intrinsically more (top-down) motivating than the simple, reactive training used here may yield significant additional benefit (Amitay et al. 2010).

Frequency discrimination training, for most individuals leads to measurable perceptual learning, and for some appears to have benefit in terms of a small reduction in tinnitus handicap. However, this is not specifically associated with compensatory sound enrichment: thus, we do not report any major significant differences between five groups of participants here. The current study also does not allow us to conclusively identify participant characteristics that would indicate training as a useful intervention for a given individual, although those with less intrusiveness tinnitus at baseline appeared to fare best. Further indicators for the efficacy of the intervention may come from studies that concentrate efforts on training larger cohorts of individuals on the same stimulus. Given the effects reported here, future studies should also include a control group who perform a task other than frequency discrimination training, or who receive a form of passive sound stimulation only. Given strong evidence for the role of lateral inhibition in tinnitus generation, it would be of great interest to test for any differential effects of training groups of individuals matched for their tinnitus spectra using various frequencies outside the spectrum of their tinnitus and hearing loss. Future work should concentrate on optimising the efficacy of training by delivering it in different, potentially more engaging formats and over longer-term studies, before exploring its physiological mechanisms of action.

## Electronic supplementary material

Below is the link to the electronic supplementary material.

Mean values and 95 % confidence intervals for THQ and THQ factors 1 and 2 scores (JPEG 80 kb)

High-resolution image (TIFF 1,073 kb)
