# Supplementary figures and images for: Effects of Frequency Discrimination Training on Tinnitus: Results from Two Randomised Controlled Trials
Source: J Assoc Res Otolaryngol. 2012 Apr 4;13(4):543–59. doi: 10.1007/s10162-012-0323-6 (PMC3387303; doi:10.1007/s10162-012-0323-6)

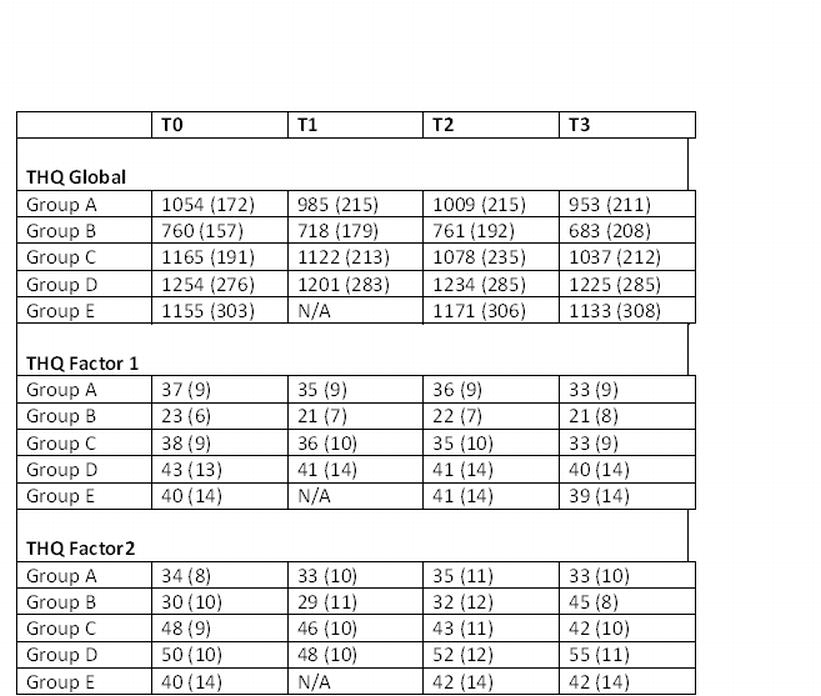

Supplement: Supplementary file 1 — Mean values and 95 % confidence intervals for THQ and THQ factors 1 and 2 scores (JPEG 80 kb) [file 10162_2012_323_Fig6_ESM.jpg]

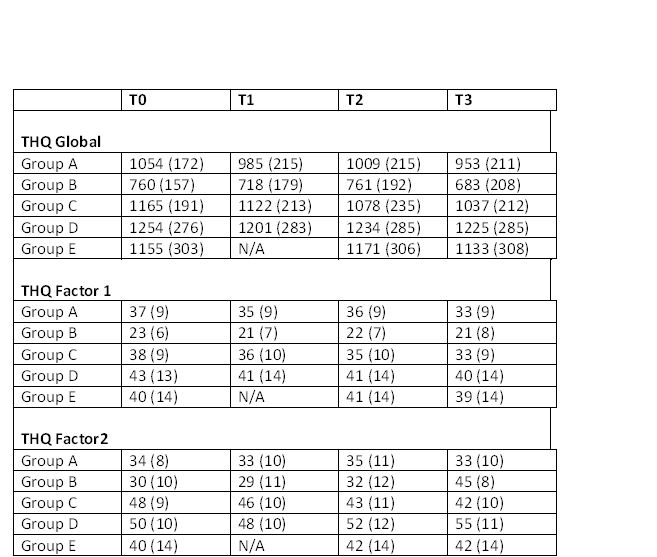

Supplement: Supplementary file 2 — High-resolution image (TIFF 1,073 kb) [file 10162_2012_323_MOESM1_ESM.tif]
